# Erratum for “Evaluation of compounded trilostane packets for dogs with naturally occurring hyperadrenocorticism”

**DOI:** 10.1111/jvim.16608

**Published:** 2022-12-09

**First published: 10 June 2021** https://onlinelibrary.wiley.com/doi/10.1111/jvim.16197

**Volume 35, Issue 4**

Correction to the grant/award number for the funder as follows.

**Funding information:** Rural Development Administration, Grant/Award Number: PJ01404502.

